# Associations between home gardening and obstructive sleep apnoea: role of behavioural factors in the COMmunity-based Behaviour and Attitude Study in Tuvalu (COMBAT)

**DOI:** 10.7189/jogh.15.04225

**Published:** 2025-08-15

**Authors:** Chia-Chen Lin, Po-Jen Lin, Tai-Lin Lee, Stephanie M Wu, Chih-Wei Shih, Selotia Tausi, Vine Sosene, Pauke P Maani, Malo Tupulaga, Shi-Chian Shiau, Yuan-Hung Lo, José Francisco López-Gil, Maria Soledad Hershey, Chia-Rui Chang, Yu-Tien Hsu, Chih-Fu Wei

**Affiliations:** 1 Department of Otorhinolaryngology and Head and Neck Surgery, New Taipei Municipal Tucheng Hospital, New Taipei City, Taiwan; 2 Department of Medicine, Nuvance Health Danbury Hospital, Danbury, Connecticut, USA; 3 Taiwan International Cooperation and Development Fund (ICDF), Taipei, Taiwan; 4 Taiwan Technical Mission to Tuvalu, Funafuti, Tuvalu; 5 Department of Radiology and Imaging Science, Emory University School of Medicine, Atlanta, Georgia, USA; 6 Department of Biostatistics, Harvard T.H. Chan School of Public Health, Boston, Massachusetts, USA; 7 Department of Agriculture, Funafuti, Tuvalu; 8 Department of Public Health, Ministry of Health, Funafuti, Tuvalu; 9 School of Medicine, Universidad Espíritu Santo, Samborondón, Ecuador; 10 Vicerrectoría de Investigación y Postgrado, Universidad de Los Lagos, Osorno, Chile; 11 Human Flourishing Program, Institute for Quantitative Social Science, Harvard University, Cambridge, Massachusetts, USA; 12 Center for Biostatistics in AIDS Research, Harvard T.H. Chan School of Public Health, Boston, Massachusetts, USA; 13 Department of Social and Behavioural Sciences, Yale School of Public Health, New Haven, Connecticut, USA; 14 Department of Environmental and Occupational Medicine, National Taiwan University Hospital Yunlin Branch, Yunlin, Taiwan; 15 Department of Environmental and Occupational Medicine, National Taiwan University College of Medicine and Hospital, Taipei, Taiwan

## Abstract

**Background:**

Tuvalu, like many Pacific Island nations, is facing a severe obesity epidemic, which is strongly associated with obstructive sleep apnoea (OSA) – a condition linked to multiple health complications and a growing public health burden. Lifestyle interventions such as home gardening have emerged as potential strategies to address obesity and its related conditions. We investigated the association between home gardening and OSA risk in Tuvalu and explored how behavioural and demographic factors may modify this relationship.

**Methods:**

We conducted a nationwide cross-sectional study in Tuvalu in 2023 using the COMmunity-based Behaviour and Attitude Study in Tuvalu (COMBAT) questionnaire. We assessed OSA risk using the validated eight-item ‘Snoring, Tiredness, Observed apnoea, high blood Pressure, Body mass index, Age, Neck circumference, and Gender’ (STOP-Bang) questionnaire, and home gardening status was self-reported. We used logistic regression models, including multivariable adjustments and overlap weighting, to assess the association between home gardening and OSA outcomes, including total STOP-Bang score, OSA risk (≥3 points), and related symptoms (snoring, daytime fatigue, witnessed apnoea). We conducted stratified analyses by behavioural and demographic characteristics.

**Results:**

We included 849 adult participants (mean (x̄) age = 32.9 years; 51.9% female). Among individuals who exercised, home gardening was associated with lower STOP-Bang scores (x̄ difference = –0.30; 95% confidence interval (CI) = –0.59, –0.01, *P* = 0.040 in overlap weighting model) and with lower odds of STOP-Bang score ≥3 (adjusted odds ratio = 0.85; 95% CI = 0.73, 0.98, *P* = 0.026) than non-gardeners. Furthermore, home gardening was associated with significantly lower odds of snoring among individuals who smoked, consumed alcohol, or exercised, and with borderline lower odds of STOP-Bang score ≥3 among individuals who consumed alcohol.

**Conclusions:**

In the COMBAT study, home gardening was associated with lower OSA probability among subgroups with specific lifestyle factors. These findings suggest that home gardening could serve as a feasible and community-based intervention to mitigate OSA risk in Tuvalu and similar low-resource settings.

Obstructive sleep apnoea (OSA) is an increasingly common sleep disorder characterised by recurrent upper airway obstruction during sleep, leading to intermittent hypoxia and sleep fragmentation [1–4]. A 2019 global study estimated that approximately one billion adults aged 30–69 years have OSA, with approximately half experiencing moderate to severe symptoms [2]. Despite its high prevalence, most cases remain undiagnosed and untreated, especially among women [5–9]. Untreated OSA is associated with significant health risks, including cardiovascular disease, metabolic disorders, and increased risk of traffic accidents, mortality, and sudden death [10–12]. The pathogenesis of OSA is multifactorial, involving obesity, the primary risk factor, as well as local tissue hypertrophy and lifestyle factors such as insufficient physical activity, unhealthy diet [1,13–17], and environmental exposures such as air pollution and greenspace [18,19]. The rising global obesity epidemic has heightened concerns about OSA, particularly in the Western Pacific region [20–22]. In Tuvalu, a small South Pacific Island nation where the prevalence of obesity reaches approximately 70% of women and 50% of men, OSA may be a significant yet underrecognized health burden [22,23]. Continuous positive airway pressure is an effective OSA intervention, but has limited availability in Tuvalu [24]. Given the high prevalence and serious consequences of untreated OSA, increased awareness, screening, early diagnosis, and targeted intervention strategies addressing its risk factors could help mitigate the impact of OSA and its associated complications in Tuvalu.

Home gardening promotes a healthy lifestyle that may reduce the probability of having OSA [25–28], and a previous study conducted by our research team revealed that it was associated with a lower probability of obesity in Tuvalu [29]. Gardening promotes physical activity, exposure to green space, and a healthier diet, which may help reduce OSA risk [13,19,30–32]. Activities during gardening could enhance physical fitness, lung function, and respiratory health [32]. Additionally, these behaviours regulate circadian rhythms, lower stress hormone levels (*i.e.* cortisol) [33], and improve mood and sleep quality [25,28,31,32,34–36]. Home gardening has also been associated with a healthier diet [26,31,37]. Moreover, gardeners are more likely to meet dietary recommendations by consuming more fresh fruits and vegetables, which supports weight management and metabolic health [38]. This is particularly relevant in Tuvalu, where high obesity prevalence, climate change, and limited access to fresh produce increase the probabilities of having OSA [23,39–41]. Our previous results showing a lower probability of having severe obesity, especially in Funafuti, the main island of Tuvalu, indicated a promising lifestyle intervention with home gardening [23]. Despite these potential benefits, the prevalence and clinical impact of OSA in Tuvalu remain unclear. Research is needed to assess the effects of home gardening on OSA and identify populations that may benefit most from such intervention. Therefore, this study aimed to examine the association between home gardening and the prevalence of OSA via a validated assessment tool and related symptoms. We also sought to identify subgroups that may benefit the most through stratified analysis.

## METHODS

### Study population

This study is a secondary data analysis study of the Community-based Behaviour and Attitude Study in Tuvalu (COMBAT), a nationwide administrative survey examining health conditions and related risk factors in Tuvalu under the context of climate change. Since 2011, the Tuvalu government and Taiwan Technical Mission have collaborated on the Tuvalu Fruit and Vegetable Production and Nutrition Enhancement Project to improve community health through fruit and vegetable cultivation [42]. In 2023, we carried out a survey in Funafuti and Vaitupu to assess agricultural practices and lifestyle factors that could promote public health and environmental sustainability in Tuvalu. Funafuti is the main island of Tuvalu, where half of the Tuvalu population lives across the seven villages on Fongafale islet (Fakaifou, Senala, Alapi, Vaiaku, Lofeagai, Teone, and Tekavatoetoe). Meanwhile, Vaitupu is the second largest island in Tuvalu, where the main residential area is around the island wharf. We estimated that there would be power to detect a risk factor for obesity with an odds ratio of 1.5 with α = 0.05 and *β* = 0.2 with a sample size of n = 900 based on our previous findings [23]. We used convenience sampling methods at community gathering locations to facilitate efficient recruitment, given the absence of civilian personal identification numbers and address systems in Tuvalu [23,43].

We developed a questionnaire to collect information on demographics, health behaviours, home garden use, biometrics, and health-related factors (Appendix S1 in the **Online Supplementary Document**). We previously trained local interviewers, and pilot tests of the questionnaire were conducted at the Taiwan Technical Mission to ensure the questionnaire’s applicability. The local research team conducted face-to-face interviews in Funafuti villages and Vaitupu wharf, and measured height, waist circumference, and neck circumference with a tape measure in cm and weight with an electronic scale in kg. The data collection via in-person interviews took place between February–May 2023, with prior approval from the Tuvalu Ministry of Health (Appendix S2 in the **Online Supplementary Document**). All participants provided written informed consent before the study began.

### Outcomes of interest

We measured OSA risk via the ‘Snoring, Tiredness, Observed apnea, high blood Pressure, Body mass index, Age, Neck circumference, and Gender’ (STOP-Bang) questionnaire, a widely used screening tool for OSA [44]. The STOP-Bang score incorporates body mass index (BMI) (defined as body weight divided by height-square, ≥35 kg/m^2^ or not), age (≥50 years old or not), neck circumference (≥40 cm or not), sex (male or female), history of high blood pressure (yes or no), snoring (yes or no), daytime fatigue (yes or no), and witnessed apnoea (yes or no). Each item in the STOP-Bang score is one point, with total scores ranging from zero to eight points. Higher scores indicate a greater risk of OSA, and we set a STOP-Bang score of three points as the cutoff point between lower and higher OSA risk. A STOP-Bang score of ≥3 is highly sensitive for detecting all OSA severities, ranging from 81% in South Asia or Southeast Asia to 95% in Europe [8]. We also modelled OSA-related symptoms (snoring, daytime fatigue, and witnessed apnoea) as binary outcomes.

### Exposure of interest

The participants reported their home garden use status through a single question during their interviews: ‘Does your family own a home garden?,’ which was ‘*E isi se otou fatoaga ite fale (Ao or Ikai)?*’ in the Tuvaluan version. We coded the response as a binary variable (yes or no). We also asked the participants about the size of their home gardens (‘*Pefea te lasi o te fatoaga?*’ in m^2^), the main caretaker of the home gardens (‘*Kooi a tino e paanaki saale mo latou te tokiga/tausiga o te fatoaga?* which responded as self, parents, children or other family members), and the crops grown in the home garden (‘*Nea fuaga lakau/vesiapolo e toki ite fatoaga (nei io me se taimi ko teka atu)?*’ with responses as seven common crops in Tuvalu or others) during the interview. The study presented the characteristics of home gardens in Tuvalu (Table S1 in the **Online Supplementary Document**).

### Covariates of interest

We selected covariates on the basis of background knowledge of home garden use and health in Tuvalu, with input from local experts and interviewers. These covariates included age (years), sex (female or male) [45], weekly exercise time (in minutes) [1,15], education level (college or above *vs.* others), work status (regular/fixed work, temporary work, or none/students), current residence (Funafuti or Vaitupu) [46], smoking (yes, defined as every day, occasional, or social use; or no), alcohol consumption (yes, defined as every day, occasional, or social use; or no) and past medical history of noncommunicable diseases (defined as having self-reported, physician-diagnosed history of hypertension, diabetes or dyslipidaemia, yes or no) [1,13,16,21].

### Statistical analysis

We summarised the baseline characteristics of the study population via descriptive statistics and the health-related parameters (**Table 1**, **Table 2**). We examined the normality of continuous variables with the quantile-quantile plot. Characteristics are presented as the means and standard deviations for continuous variables with normal distributions, and percentages for categorical variables, according to home garden use.

**Table 1:** Baseline characteristics of participants from the Tuvalu Fruit and Vegetable Production and Nutrition Enhancement Project*

Characteristics	Home garden users (n = 108)	Home garden non-users (n = 741)	*P*-value
Age in years, x̄ (SD)	36.0 (13.8)	32.4 (12.6)	0.007
Female	55 (50.9)	386 (52.1)	0.902
Vaitupu residence	14 (13.0)	132 (17.8)	0.266
Duration of residence on current island in years, x̄ (SD)	15.6 (12.0)	15.6 (10.8)	0.944
Income in AUD per month, x̄ (SD)	248.2 (368.4)	132.8 (331.9)	0.001
Marital status			0.482
*Single*	33 (30.6)	260 (35.1)	
*Married*	73 (67.6)	454 (61.4)	
*Widowed*	2 (1.9)	18 (2.4)	
*Divorced*	0 (0.0)	8 (1.1)	
Education level			0.001
*None*	10 (9.3)	174 (23.5)	
*Elementary school*	15 (13.9)	63 (8.5)	
*High school*	40 (37.0)	295 (39.8)	
*College or above*	43 (39.8)	209 (28.2)	
Employment status			0.007
*Regular/fixed work*	32 (29.6)	153 (20.6)	
*Temporary work*	20 (18.5)	89 (12.0)	
*None/students*	56 (51.9)	499 (67.3)	
Weekly exercise time in minutes, x̄ (SD)	115.4 (205.4)	84.8 (160.5)	0.075
Smoking	32 (29.6)	234 (31.6)	0.766
Second-hand smoke exposure	69 (63.9)	418 (56.4)	0.173
Alcohol consumption	35 (32.4)	268 (36.2)	0.513

SD – standard deviation, x̄ – mean

*Presented as n (%) unless specified otherwise.

**Table 2:** Descriptive statistics for health parameters among home garden users and non-users*

Items	Home garden users (n = 108)	Home garden non-users (n = 741)	*P*-value
STOP-Bang score, x̄ (SD)	2.8 (1.4)	2.7 (1.2)	0.693
STOP-Bang score ≥3	54 (50.0)	381 (51.4)	0.863
Body mass index in kg/m^2^, x̄ (SD)	33.7 (6.9)	33.4 (7.1)	0.674
Waist circumference in cm, x̄ (SD)	95.9 (16.7)	92.0 (17.3)	0.028
Neck circumference in cm, x̄ (SD)	38.3 (4.7)	38.3 (4.9)	0.895
Observed symptoms			
*Witnessed apnoea*	17 (15.7)	85 (11.5)	0.264
*Snoring episode*	27 (25.0)	213 (28.7)	0.488
*Daytime fatigue*	31 (28.7)	255 (34.4)	0.287
Non-communicable disease diagnosis			
*Hypertension*	18 (16.7)	86 (11.6)	0.180
*Diabetes mellitus*	8 (7.4)	52 (7.0)	1.000

SD – standard deviation, x̄ – mean

*Presented as n (%) unless specified otherwise.

**Figure 1:**
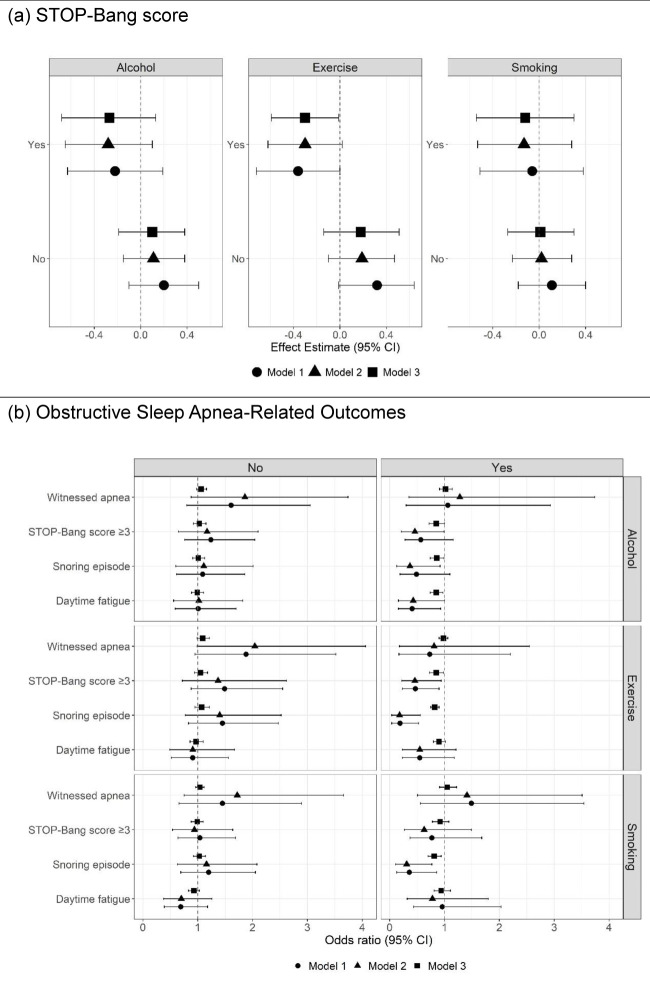
Unadjusted, adjusted, and weighted associations of home gardening with STOP-Bang score, and obstructive sleep apnoea-related outcomes, stratified by behavioural factors.

We tested the associations between home garden use and the STOP-Bang score, increased OSA risk (STOP-Bang score ≥3), snoring, daytime fatigue, and witnessed apnoea via univariate and multivariate linear and logistic regression. Additionally, we applied an overlap weighting approach to address confounding, which assigns weights to individuals on the basis of their probability of being in the opposite group. This method assigns greater weight to patients with similar probability of receiving either treatment while reducing the influence of those at the extremes of the propensity score distribution. Compared to standard inverse probability weighting, this method improves balance and precision compared with standard inverse probability weighting by reducing the impact of extreme weights [47,48]. We tested the associations in the overall population (**Table 3**) and stratified them by exercise level, smoking status, and alcohol use (yes/no) (**Figure 1**; Table S2 in the **Online Supplementary Document**). We also tested the associations in the populations stratified by demographic and socioeconomic status, including sex, residence, education level and noncommunicable disease diagnosis (*i.e.* self-reported history of hypertension, diabetes and dyslipidaemia) (Table S3 in the **Online Supplementary Document**). In these stratified analyses, the stratified variables were excluded from the regression model to avoid model fit issues caused by single-level covariates.

**Table 3:** Unadjusted, adjusted, and weighted associations of home gardening with obstructive sleep apnoea outcomes in the COMBAT population

Home garden users *vs.* non-users	Unadjusted		Adjusted*		Overlap weighting	
	**Estimate (95% CI)**	***P*-value**	**Estimate (95% CI)**	***P*-value**	**Estimate (95% CI)**	***P*-value**
STOP-Bang score	0.05 (–0.20, 0.29)	0.693	–0.03 (–0.25, 0.18)	0.783	–0.04 (–0.27, 0.19)	0.735
STOP-Bang score ≥3	0.94 (0.63, 1.42)	0.783	0.81 (0.51, 1.28)	0.366	0.96 (0.87, 1.05)	0.347
Snoring episode	0.83 (0.51, 1.30)	0.420	0.78 (0.46, 1.27)	0.325	0.96 (0.88, 1.04)	0.323
Daytime fatigue	0.77 (0.49,1.18)	0.242	0.75 (0.46, 1.20)	0.244	0.94 (0.86, 1.03)	0.158
Witnessed apnoea	1.44 (0.80,2.48)	0.204	1.54 (0.82, 2.75)	0.159	1.04 (0.97, 1.12)	0.238

CI – confidence interval, STOP-Bang – Snoring, Tiredness, Observed apnoea, Pressure (high blood pressure), Body Mass Index, Age, Neck circumference, and Gender

*Adjusted for age (years), gender (male or female), education (college or above *vs.* others), region (Funafuti *vs.* others), self-reported non-communicable disease diagnosis (yes or no), alcohol consumption (yes or no), smoking (yes or no) and exercise time per week (minutes).

This study also reports associations between behavioural and demographic factors and obstructive sleep apnoea outcomes, including STOP-Bang scores, STOP-Bang scores ≥3, snoring episodes, daytime fatigue, and witnessed apnoea (Table S4 in the **Online Supplementary Document**). We considered two-sided *P*-values <0.05 statistically significant. We conducted all analyses with *R*, version 4.2.3 (R Core Team, Vienna, Austria).

## RESULTS

### Descriptive characteristics of the study population

During the study period, we included 890 Tuvaluan citizens aged ≥18 years who provided written informed consent before participating in the study. We excluded 41 participants with missing data for the outcomes of interest (n = 29), exposure of interest (n = 3), and main demographic and behavioural factors (n = 9). As a result, we included 849 COMBAT participants in the analysis, of which 108 (12.7%) participants reported home garden use.

The home garden users were older (aged 36.0 *vs.* 32.4 years, *P* = 0.007), had higher incomes (AUD 248.2 *vs.* AUD 132.8 per month, *P* = 0.002), had a higher education level (*P* = 0.001), and were more likely to have a regular employment status (*P* = 0.007) than nonusers. The sex distribution was balanced, with slightly more women included (50.9% among home garden users and 52.1% among nonusers, *P* = 0.902). The proportion of respondents from the outlying island (Vaitupu) was similar between home garden users (13.0%) and non-users (17.8%), as was the duration of residence on each island. Home garden users had a non-significantly higher mean weekly exercise time (115.4 *vs.* 84.8 minutes, *P* = 0.075). Moreover, there were no significant differences in marital status, duration of residence on the current island, smoking status, alcohol consumption, or second-hand smoke exposure between home garden users and nonusers (**Table 1**).

There were no significant differences in the STOP-Bang score, BMI, neck circumference, observed symptoms, or noncommunicable disease diagnosis. Interestingly, the average waist circumference of home garden users was slightly greater than that of nonusers (95.9 *vs.* 92.0 cm, *P* = 0.028) (**Table 2**).

### Behavioural factors modified the association between home garden use and obstructive sleep apnoea risk in Tuvalu

Home garden use was not associated with OSA-related outcomes in the total population; however, associations emerged in the exploratory stratified analyses by behavioural factors (**Figure 1**, **Table 3**). Adjusted and overlap weighting analyses revealed significant differences in the STOP-Bang score, STOP-Bang score ≥3, snoring episode, and daytime fatigue between home garden users and nonusers in several subgroups in overlap weighting models. Among those with exercise habits, home garden users had a 0.30-point lower STOP-Bang score (95% confidence interval (CI) = –0.59, –0.01, *P* = 0.040), a lower likelihood of having a STOP-Bang score ≥3 (adjusted odds ratio (aOR) = 0.85; 95% CI = 0.77, 0.98, *P* = 0.026) and a lower likelihood of experiencing snoring (aOR = 0.82; 95% CI = 0.75, 0.90, *P* < 0.001) than nonusers. Home gardening was also associated with lower odds of STOP-Bang score ≥3 among those who consumed alcohol (aOR = 0.85; 95% CI = 0.72, 1.00, *P* = 0.047), and lower odds of snoring (aOR = 0.81; 95% CI = 0.70, 0.94, *P* = 0.005 among individuals who smoked, aOR = 0.86; 95% CI = 0.74, 0.99, *P* = 0.034 among individuals consumed alcohol, and aOR = 0.82; 95% CI = 0.75, 0.90, *P* < 0.001 among individuals with exercise habits). Meanwhile, no significant association of home gardening was observed among individuals who did not exercise, smoke, or consume alcohol.

### Demographic factors and the association between home garden use and obstructive sleep apnoea risk in Tuvalu

No significant differences were observed in these associations by sex, island of residence (Funafuti or Vaitupu), education level (college or above, or others), or noncommunicable disease status (yes or no) for all OSA-related outcomes in this study, including the STOP-Bang score, STOP-Bang score ≥3, snoring episode, observed OSA episodes and daytime fatigue (Table S2 in the **Online Supplementary Document**).

### Sensitivity analysis

Histories of smoking and of noncommunicable disease diagnosis were associated with higher STOP-Bang scores and increased odds of having STOP-Bang scores ≥3, snoring episodes, daytime fatigue and witnessed apnoea. In contrast, being female, having an education level of college or above, and living outside of Funafuti were associated with lower STOP-Bang scores and lower odds of the above outcomes. We did not observe significant associations for alcohol use or exercise time (Table S3 in the **Online Supplementary Document**).

## DISCUSSION

### Summary of findings

In this study, we revealed that the association between home gardening and the probability of having OSA varied by an individual’s behavioural factors. Home gardening was associated with lower STOP-Bang scores, lower odds of STOP-Bang score ≥3 and snoring among individuals with exercise habits. Home gardening was associated with lower odds of STOP-Bang score ≥3 and snoring among alcohol consuming individuals, and lower odds of STOP-Bang score ≥3 among smokers. No significant association of home gardening was observed among non-smokers, alcohol non-consumers, and participants without exercise habits. These findings suggest a viable hypothesis that these behavioural factors may modify the relationship between home gardening and the probability of having OSA, indicating home gardening may be a particularly beneficial lifestyle intervention for those with exercise habits and people with greater OSA risk. Meanwhile, the association between home gardening and OSA-related outcomes varied little by demographic factors such as sex, island of residence, education level, and noncommunicable disease status.

### Associations between home gardening and OSA probability: roles of exercise, alcohol use, and smoking

We found an inverse association between home gardening and OSA prevalence and related symptoms among individuals with exercise habits, suggesting that integrating gardening into their relatively more active lifestyle may further enhance its protective effects against OSA. A study in Australia showed that regular exercise could significantly reduce OSA severity by promoting favourable fat distribution even without substantial weight loss [49]. Home gardening involves multiple low- to moderate-intensity activity tasks with metabolic equivalent of task values between 1.7–4.5, such as planting and harvesting, which support an active lifestyle [36]. The benefits of increased physical activity extend beyond reducing the apnoea – hypopnea index to improving sleep quality [14], better physical fitness, lung function, and respiratory health [28,36]. Our previous study in the same Tuvalu population also revealed that home gardening was associated with a lower probability of obesity, which supports the above argument [29]. These findings supported that home gardening may reduce the probability of having OSA and experiencing related symptoms, possibly through additional physical activity that showed more pronounced health benefits among those with exercise habits [1,15,32]. In Tuvalu, incorporating home gardening into exercise promotion programs may be a promising strategy, as it not only encourages regular physical activity but also provides a sustainable source of fruits and vegetables.

The stronger association between home gardening and lower probability of having OSA in smokers and alcohol users may be related to greater greenspace exposure and increased vegetable intake [31]. Home gardening increases neighbourhood greenspace exposure and supports smoking cessation, which may provide additional health benefits to lower OSA risk for current smokers [19,50]. A study based on the Canadian Community Health Survey (2016–18) showed that residential greenness was associated with lower odds of tobacco use and binge drinking, supporting that home gardening may also reduce smoking and alcohol consumption through increased greenspace exposure [51]. The benefits of greenspace exposure are supported by another study using Amazon Mechanical Turk data, which revealed that actively spending time in nature was associated with a lower negative effect and greater positive affect against problematic alcohol use [52]. At the same time, a study in Australia revealed that those involved in gardening facilitate vegetable consumption, which could counteract the adverse health effects caused by smoking and alcohol use [53]. Furthermore, a quasi-experimental study among 224 control and 395 households with home garden interventions in rural Bangladesh revealed that participants in the home garden intervention group had higher vegetable production and improved supply of micronutrients, including iron, zinc, folate, and provitamin A [27]. The authors also found a sustained increase in women’s nutritional knowledge and gardening practices over the three-year study period, which may explain in part such behavioural changes [27]. In summary, home gardening can be a sustainable intervention to existing alcohol and smoking cessation programs, given the reduced OSA risk, benefits of greenspace exposure and higher micronutrient intake from vegetables.

### Advocating home gardening across diverse sociodemographic groups

Although the associations stratified by sex, residence, education level, or noncommunicable disease status were not significant, the direction of associations suggested a lower probability of OSA among home gardeners. In our study, males showed higher STOP-Bang scores and a greater likelihood of snoring, which aligned with the existing gender differences in OSA risk, with approximately twice the incidence of OSA in men than in women [21,45]. The physiological differences, including body composition, hormonal profiles, and susceptibility to environmental exposure, may influence the observed association between home gardening and OSA risk in certain subgroups [1,6,7]. For example, men are more likely to have classic signs such as snoring, whereas women often present with atypical symptoms such as insomnia or morning headaches, making OSA frequently underdiagnosed among women [1,6,54]. Moreover, men and women have different perspectives and involvement in home gardening. A study in Malaysia and Egypt revealed that men and women had different perspectives on the relationship between gardening activities and their possible benefits, and home gardening participation may be influenced by traditional gender roles and societal structures [55]. Women may face barriers to engaging in such activities, as was suggested in a Bangladesh population, inhibiting their potential health benefits [27]. In Tuvalu society, domestic role limitations may affect women's participation in home gardens, and further research is needed to develop gender-aware interventions, especially for populations with greater responses.

Promoting home gardening is important both in Funafuti and the outer islands. In our previous research, residents of the outer islands were more likely to have home gardens than were Funafuti residents, according to COMBAT data [46]. This disparity is partly due to limited land availability in the densely populated Funafuti, where many residents rent their homes, in contrast with the more spacious outer islands [46]. Despite these differences, our study revealed that home gardening offers potential health benefits across all regions of Tuvalu, which highlights the need to promote home gardening throughout the country to advance public health. Overall, our findings are consistent with the literature regarding home gardening, which offers numerous health benefits, possibly through improved nutrition, increased physical activity, and community support [25,34,35].

### Strengths and limitations

There are several strengths in this study. The standardised screening tool for OSA that was used, the STOP-Bang, is suitable for resource-limited settings. Systematic training for neck circumference measurements was conducted to ensure consistency in the data collection. A structured questionnaire was used to collect demographic and behavioural information, and overlap weighting was applied in this study to minimise bias. In contrast, some limitations are worth mentioning. The STOP-Bang questionnaire has limited specificity, and the prevalence of snoring and other symptoms may be underestimated in the self-reported design. Therefore, future studies should incorporate objective diagnostic tools such as a portable polysomnography to confirm OSA diagnosis. We applied a convenience sampling scheme, and individuals with the highest risk for OSA or who were hospitalised may have been underrepresented in COMBAT, which could affect the generalizability of our findings. We excluded participants without information of OSA and home gardening, which could introduce information bias in this study. Recall bias and social desirability bias may affect self-reported symptoms and gardening habits. Additionally, the cross-sectional design of this study hinders the ability to draw causal inferences. Future research employing longitudinal designs is warranted to establish temporal relationships and infer causality. Finally, although we applied multivariable adjustment and overlap weighting, the potential for unmeasured confounders remains.

## CONCLUSIONS

Home gardening was associated with a lower likelihood of OSA and related symptoms among individuals with exercise habits, smokers, and alcohol consumers in Tuvalu. These findings underscore the importance of identifying target populations that may benefit the most from this intervention, particularly those with lifestyle-related risk factors for OSA. Future research should investigate the mechanisms underlying these associations, assess the long-term impact of home gardening on sleep health, and evaluate its potential effect as a targeted preventive measure for high-risk groups. Home gardening may help foster healthier lifestyles and enhance overall community well-being. Furthermore, home gardening may reduce the burden of OSA as a lifestyle prevention strategy when combined with existing public health campaigns of exercise promotion, smoking and alcohol cessation, particularly in resource-limited settings.

## Additional material


Online Supplementary Document
