# Supplementary material for: Associations between home gardening and obstructive sleep apnoea: role of behavioural factors in the COMmunity-based Behaviour and Attitude Study in Tuvalu (COMBAT)
Source: J Glob Health. 2025 Aug 15;15:04225. doi: 10.7189/jogh.15.04225 (PMC12355442; doi:10.7189/jogh.15.04225)
Supplement: Online Supplementary Document [file jogh-15-04225-s001.pdf]

Supplement to: Lin C, Lin P, Lee T, Wu SM, Shih C, Tausi S, Sosene V, Maani PP, Tupulaga M, Shiau S, Lo Y, López-Gil JF, Hershey MS, Chang C, Hsu Y, Wei C. Associations between home gardening and obstructive sleep apnoea: role of behavioural factors in the COMmunity-based Behaviour and Attitude Study in Tuvalu (COMBAT). J Glob Health. 2025;15:04225.

Table S1. Characteristics of home gardens in the study population, presented in mean ± SD or N (%)

	**Home garden users (n=108)**
Home garden size (Square meters)	7.7 ± 7.0
Main caretaker of the home garden (%)	
Self	29 (32.2)
Parents	27 (30.0)
Children	5 (5.6)
Other family members	29 (32.2)
Crops (%)	
Spinach	5 (4.6)
Pepper (capsicum, chili)	52 (48.1)
Cabbage	88 (81.5)
Cucumber	76 (70.4)
Tomato	66 (61.1)
Pumpkin	18 (16.7)
Others	66 (61.1)

SD – standard deviation

Table S2. Unadjusted, adjusted, and weighted associations of home gardening with obstructive sleep apnea outcomes, stratified by behavioral factors

	**Unadjusted**	**Adjusted**	**Overlap weighting**
	**Estimate (95% CI, p-value)**	**Estimate (95% CI, p-value)**	**Estimate (95% CI, p-value)**
People with exercise habit *			
STOP-Bang score	-0.36 (-0.72-0.00, p=0.052)	-0.30 (-0.62-0.02, p=0.067)	-0.30 (-0.59--0.01, p=0.040)
STOP-Bang score ≥3	0.47 (0.23-0.90, p=0.026)	0.46 (0.22-0.94, p=0.035)	0.85 (0.73-0.98, p=0.026)
Snoring episode	0.19 (0.04-0.53, p=0.006)	0.18 (0.04-0.56, p=0.008)	0.82 (0.75-0.90, p=0.000)
Daytime fatigue	0.55 (0.23-1.18, p=0.147)	0.55 (0.23-1.21, p=0.160)	0.90 (0.80-1.02, p=0.098)
Witnessed apnea	0.73 (0.17-2.20, p=0.619)	0.81 (0.18-2.55, p=0.741)	0.98 (0.90-1.06, p=0.582)
No exercise habit *			
STOP-Bang score	0.32 (-0.01-0.64, p=0.058)	0.19 (-0.10-0.47, p=0.195)	0.18 (-0.14-0.51, p=0.266)
STOP-Bang score ≥3	1.49 (0.88-2.55, p=0.141)	1.37 (0.72-2.62, p=0.341)	1.05 (0.94-1.18, p=0.351)
Snoring episode	1.45 (0.83-2.47, p=0.181)	1.40 (0.77-2.52, p=0.261)	1.07 (0.95-1.21, p=0.283)
Daytime fatigue	0.91 (0.52-1.56, p=0.748)	0.91 (0.49-1.67, p=0.767)	0.97 (0.86-1.10, p=0.656)
Witnessed apnea	1.88 (0.95-3.52, p=0.059)	2.04 (0.99-4.06, p=0.047)	1.09 (0.99-1.21, p=0.085)
Smokers †			
STOP-Bang score	-0.06 (-0.51-0.38, p=0.779)	-0.13 (-0.53-0.28, p=0.538)	-0.12 (-0.54-0.30, p=0.579)
STOP-Bang score ≥3	0.77 (0.37-1.68, p=0.506)	0.63 (0.27-1.49, p=0.286)	0.92 (0.78-1.08, p=0.313)
Snoring episode	0.36 (0.13-0.86, p=0.032)	0.31 (0.11-0.77, p=0.018)	0.81 (0.70-0.94, p=0.005)
Daytime fatigue	0.96 (0.44-2.03, p=0.916)	0.78 (0.32-1.80, p=0.574)	0.94 (0.81-1.11, p=0.484)
Witnessed apnea	1.49 (0.56-3.54, p=0.389)	1.41 (0.51-3.51, p=0.479)	1.05 (0.91-1.22, p=0.475)
Non-smokers †			
STOP-Bang score	0.11 (-0.18-0.40, p=0.446)	0.02 (-0.23-0.28, p=0.860)	0.01 (-0.27-0.30, p=0.939)
STOP-Bang score ≥3	1.04 (0.64-1.69, p=0.860)	0.94 (0.54-1.64, p=0.831)	0.99 (0.88-1.10, p=0.819)

Snoring episode	1.20 (0.69-2.05, p=0.501)	1.16 (0.63-2.08, p=0.625)	1.03 (0.92-1.14, p=0.630)
Daytime fatigue	0.69 (0.39-1.18, p=0.189)	0.70 (0.38-1.26, p=0.252)	0.93 (0.83-1.03, p=0.181)
Witnessed apnea	1.45 (0.66-2.89, p=0.319)	1.72 (0.75-3.66, p=0.177)	1.04 (0.96-1.12, p=0.327)
Alcohol drinkers ‡			
STOP-Bang score	-0.22 (-0.63-0.19, p=0.301)	-0.28 (-0.65-0.10, p=0.144)	-0.27 (-0.68-0.13, p=0.188)
STOP-Bang score ≥3	0.57 (0.28-1.16, p=0.121)	0.46 (0.21-0.99, p=0.048)	0.85 (0.72-1.00, p=0.047)
Snoring episode	0.49 (0.19-1.10, p=0.103)	0.37 (0.13-0.92, p=0.043)	0.86 (0.74-0.99, p=0.034)
Daytime fatigue	0.41 (0.16-0.93, p=0.045)	0.43 (0.16-1.00, p=0.064)	0.85 (0.74-0.97, p=0.017)
Witnessed apnea	1.06 (0.30-2.93, p=0.914)	1.28 (0.35-3.74, p=0.673)	1.02 (0.91-1.14, p=0.763)
No alcohol consumption ‡			
STOP-Bang score	0.20 (-0.10-0.50, p=0.200)	0.11 (-0.15-0.38, p=0.395)	0.10 (-0.19-0.38, p=0.506)
STOP-Bang score ≥3	1.24 (0.76-2.04, p=0.386)	1.17 (0.65-2.10, p=0.591)	1.03 (0.92-1.15, p=0.627)
Snoring episode	1.09 (0.61-1.86, p=0.771)	1.11 (0.60-2.01, p=0.727)	1.01 (0.91-1.13, p=0.801)
Daytime fatigue	1.01 (0.59-1.70, p=0.957)	1.02 (0.56-1.82, p=0.951)	0.99 (0.89-1.11, p=0.912)
Witnessed apnea	1.61 (0.80-3.05, p=0.156)	1.86 (0.88-3.74, p=0.092)	1.06 (0.98-1.16, p=0.162)

*. Adjusted for age (years), gender (male or female), education (college or above versus others), region (Funafuti versus others), self-reported non-communicable disease diagnosis (yes or no), alcohol consumption (yes or no) and smoking (yes or no).

†. Adjusted for age (years), gender (male or female), education (college or above versus others), region (Funafuti versus others), self-reported non-communicable disease diagnosis (yes or no), alcohol consumption (yes or no) and exercise time per week (minutes).

‡. Adjusted for age (years), gender (male or female), education (college or above versus others), region (Funafuti versus others), self-reported non-communicable disease diagnosis (yes or no), exercise time per week (minutes) and smoking (yes or no).

CI – confidence interval

Table S3. Unadjusted, adjusted, and weighted associations of home gardening with obstructive sleep apnea outcomes, stratified by demographic factors

	**Unadjusted**	**Adjusted**	**Overlap weighting**	**Unadjusted**	**Adjusted**	**Overlap weighting**
	**Estimate (95% CI, p-value)**	**Estimate (95% CI, p-value)**	**Estimate (95% CI, p-value)**	**Estimate (95% CI, p-value)**	**Estimate (95% CI, p-value)**	**Estimate (95% CI, p-value)**
	Male *			*Female* *		
STOP-Bang score	0.09 (-0.24-0.42, p=0.596)	-0.01 (-0.32-0.31, p=0.962)	-0.02 (-0.39-0.36, p=0.930)	-0.01 (-0.32-0.30, p=0.952)	-0.05 (-0.35-0.25, p=0.747)	-0.05 (-0.34-0.23, p=0.722)
STOP-Bang score ≥3	0.78 (0.43-1.44, p=0.417)	0.67 (0.35-1.30, p=0.231)	0.92 (0.81-1.06, p=0.247)	1.09 (0.60-1.93, p=0.783)	0.99 (0.51-1.86, p=0.968)	1.00 (0.88-1.13, p=0.982)
Snoring episode	0.90 (0.48-1.65, p=0.743)	0.80 (0.40-1.55, p=0.518)	0.96 (0.84-1.09, p=0.525)	0.72 (0.33-1.43, p=0.375)	0.72 (0.32-1.49, p=0.398)	0.95 (0.85-1.07, p=0.399)
Daytime fatigue	0.82 (0.42-1.53, p=0.551)	0.82 (0.40-1.60, p=0.563)	0.96 (0.84-1.09, p=0.496)	0.72 (0.38-1.31, p=0.299)	0.74 (0.37-1.44, p=0.389)	0.94 (0.83-1.06, p=0.308)
Witnessed apnea	1.44 (0.59-3.14, p=0.384)	1.74 (0.68-4.04, p=0.219)	1.05 (0.95-1.17, p=0.316)	1.45 (0.63-3.03, p=0.353)	1.50 (0.62-3.36, p=0.338)	1.04 (0.95-1.14, p=0.397)
	Funafuti †			*Vaitupu* †		
STOP-Bang score	0.05 (-0.22-0.31, p=0.737)	0.05 (-0.19-0.28, p=0.702)	0.03 (-0.22-0.28, p=0.802)	0.00 (-0.62-0.62, p=0.992)	-0.35 (-0.89-0.20, p=0.211)	-0.21 (-0.71-0.28, p=0.392)
STOP-Bang score ≥3	0.80 (0.52-1.24, p=0.321)	0.78 (0.47-1.29, p=0.334)	0.95 (0.86-1.05, p=0.303)	2.44 (0.80-8.32, p=0.127)	1.59 (0.40-6.59, p=0.511)	1.16 (0.93-1.44, p=0.186)
Snoring episode	0.75 (0.44-1.23, p=0.271)	0.74 (0.42-1.27, p=0.284)	0.95 (0.87-1.04, p=0.263)	1.43 (0.42-4.42, p=0.548)	1.21 (0.32-4.15, p=0.771)	1.08 (0.82-1.44, p=0.584)
Daytime fatigue	0.67 (0.41-1.06, p=0.091)	0.72 (0.43-1.19, p=0.213)	0.93 (0.85-1.03, p=0.152)	1.73 (0.37-6.20, p=0.434)	2.00 (0.38-8.45, p=0.368)	1.07 (0.89-1.29, p=0.458)
Witnessed apnea	1.50 (0.82-2.62, p=0.166)	1.71 (0.91-3.11, p=0.086)	1.06 (0.98-1.15, p=0.125)	NA	NA	NA
	Education level at college or above ‡			*Other education levels* ‡		
STOP-Bang score	0.13 (-0.25-0.52, p=0.493)	-0.07 (-0.42-0.27, p=0.670)	-0.10 (-0.43-0.23, p=0.556)	0.10 (-0.21-0.40, p=0.536)	-0.06 (-0.33-0.22, p=0.694)	-0.06 (-0.36-0.25, p=0.706)

STOP-Bang score ≥3	0.92 (0.45-1.80, p=0.808)	0.67 (0.29-1.48, p=0.337)	0.92 (0.80-1.06, p=0.264)	1.12 (0.67-1.92, p=0.660)	0.88 (0.49-1.58, p=0.656)	0.97 (0.87-1.09, p=0.650)
Snoring episode	0.97 (0.39-2.15, p=0.935)	0.62 (0.22-1.55, p=0.336)	0.93 (0.82-1.06, p=0.277)	0.86 (0.48-1.49, p=0.592)	0.77 (0.41-1.39, p=0.394)	0.95 (0.85-1.07, p=0.423)
Daytime fatigue	0.96 (0.44-1.99, p=0.921)	0.73 (0.30-1.67, p=0.476)	0.94 (0.83-1.08, p=0.405)	0.74 (0.42-1.27, p=0.283)	0.73 (0.39-1.31, p=0.297)	0.93 (0.83-1.05, p=0.228)
Witnessed apnea	1.98 (0.61-5.61, p=0.217)	0.95 (0.23-3.26, p=0.938)	1.00 (0.91-1.09, p=0.948)	1.45 (0.71-2.76, p=0.283)	1.57 (0.75-3.12, p=0.209)	1.06 (0.96-1.17, p=0.233)
	With non-communicable disease diagnosis §			*Without non-communicable disease diagnosis* §		
STOP-Bang score	0.27 (-0.30-0.83, p=0.354)	0.03 (-0.49-0.54, p=0.923)	0.02 (-0.46-0.51, p=0.921)	-0.06 (-0.33-0.20, p=0.630)	-0.08 (-0.31-0.16, p=0.522)	-0.08 (-0.33-0.17, p=0.531)
STOP-Bang score ≥3	1.39 (0.51-4.47, p=0.541)	1.25 (0.40-4.44, p=0.716)	1.02 (0.86-1.20, p=0.848)	0.80 (0.50-1.27, p=0.351)	0.71 (0.42-1.20, p=0.206)	0.94 (0.84-1.04, p=0.209)
Snoring episode	0.84 (0.35-2.02, p=0.688)	0.81 (0.32-2.09, p=0.667)	0.94 (0.76-1.17, p=0.599)	0.70 (0.38-1.23, p=0.240)	0.75 (0.39-1.34, p=0.343)	0.96 (0.87-1.05, p=0.361)
Daytime fatigue	0.64 (0.26-1.55, p=0.319)	0.57 (0.19-1.70, p=0.309)	0.92 (0.75-1.14, p=0.439)	0.71 (0.40-1.20, p=0.216)	0.76 (0.43-1.30, p=0.330)	0.94 (0.85-1.04, p=0.260)
Witnessed apnea	2.06 (0.76-5.29, p=0.139)	2.30 (0.74-6.87, p=0.136)	1.17 (0.97-1.42, p=0.099)	1.10 (0.49-2.19, p=0.809)	1.19 (0.51-2.48, p=0.667)	1.01 (0.94-1.08, p=0.730)

*. Adjusted for age (years), education (college or above versus others), region (Funafuti versus others), self-reported non-communicable disease diagnosis (yes or no), alcohol consumption (yes or no), smoking (yes or no) and exercise time per week (minutes)

†. Adjusted for age (years), gender (male or female), education (college or above versus others), self-reported non-communicable disease diagnosis (yes or no), alcohol consumption (yes or no), smoking (yes or no) and exercise time per week (minutes). We did not provide the estimate for witnessed apnea in Vaitupu because no home garden user reported witnessed apnea.

‡. Adjusted for age (years), gender (male or female), region (Funafuti versus others), self-reported non-communicable disease diagnosis (yes or no), alcohol consumption (yes or no), smoking (yes or no) and exercise time per week (minutes)

§. Adjusted for age (years), gender (male or female), education (college or above versus others), region (Funafuti versus others), self-reported non-communicable disease diagnosis (yes or no), alcohol consumption (yes or no), smoking (yes or no) and exercise time per week (minutes)

CI – confidence interval

Table S4. Adjusted associations of behavioral and demographic factors with obstructive sleep apnea outcomes.

	STOP-Bang score	STOP-Bang score ≥3	Snoring episode	Daytime fatigue	Witnessed apnea
	**Estimate (95% CI, p-value)**	**Odds ratio (95% CI, p-value)**	**Odds ratio (95% CI, p-value)**	**Odds ratio (95% CI, p-value)**	**Odds ratio (95% CI, p-value)**
Alcohol use	-0.03 (-0.21 to 0.15, p=0.737)	1.20 (0.82-1.74, p=0.351)	1.01 (0.68-1.50, p=0.953)	1.13 (0.77-1.65, p=0.532)	0.73 (0.41-1.25, p=0.258)
Smoking	0.20 (0.03 to 0.38, p=0.021)	1.52 (1.06-2.20, p=0.025)	1.54 (1.06-2.24, p=0.022)	1.47 (1.02-2.12, p=0.040)	2.26 (1.38-3.70, p=0.001)
Exercise time (minute per week)	-0.00 (-0.00 to 0.00, p=0.338)	1.00 (1.00-1.00, p=0.319)	1.00 (1.00-1.00, p=0.799)	1.00 (1.00-1.00, p=0.196)	1.00 (1.00-1.00, p=0.053)
Female	-0.88 (-1.04 to -0.72, p<0.001)	0.28 (0.20-0.40, p<0.001)	0.64 (0.45-0.92, p=0.015)	1.41 (1.00-2.01, p=0.054)	1.15 (0.70-1.90, p=0.577)
Age (year)	0.02 (0.01 to 0.03, p<0.001)	1.04 (1.03-1.06, p<0.001)	1.00 (0.98-1.01, p=0.778)	0.98 (0.97-1.00, p=0.034)	1.00 (0.98-1.02, p=0.995)
Education level at college or above	-0.24 (-0.40 to -0.08, p=0.004)	0.62 (0.44-0.87, p=0.006)	0.67 (0.45-0.97, p=0.038)	0.67 (0.47-0.95, p=0.026)	0.52 (0.29-0.90, p=0.024)
Living outside of Funafuti	-0.27 (-0.46 to -0.08, p=0.006)	0.57 (0.38-0.86, p=0.008)	1.03 (0.67-1.56, p=0.890)	0.25 (0.15-0.41, p<0.001)	0.28 (0.12-0.59, p=0.002)
Having self-reported non-communicable disease diagnosis	0.39 (0.18 to 0.61, p<0.001)	1.94 (1.22-3.12, p=0.006)	3.97 (2.57-6.18, p<0.001)	4.70 (3.00-7.49, p<0.001)	1.99 (1.14-3.44, p=0.015)

*. Adjusted for age (years), gender (male or female), education (college or above versus others), region (Funafuti versus others), self-reported non-communicable disease diagnosis (yes or no), alcohol consumption (yes or no), smoking (yes or no) and exercise time per week (minutes)
CI – confidence interval

Savea ki luga i faifaiga masani faka-fenua i tulaga o Niutilisini, tausaga 2023

Fenua tonu/akoga	Po masina o te faka-sautalaga	Loa	‘Mafa	Waist Circumference	Neck Circumference
◯1.Funafuti; ◯2.Nanumea;<br>◯3.Nanumaga; ◯4.Niutao;<br>◯5.Vaitupu; ◯6.Nui;<br>◯7.Nukufetau; ◯8.Nukulaelae;<br>◯9. Niulakita<br>◯10.Fetuvalu High School<br>◯11.Motufoua High School	(YYYYMMDD)	(cm)	(kg)	(cm)	(cm)

Vaega I: Fesili totino	
1. Tenita	◯ 1. Tagata; ◯ 2. Fafine
2. Po fanau (DD/MM/YYYY)	
3. Avaga me seki avaga	◯ 1. Seki avaga; ◯ 2. Avaga; ◯ 3. Ko mate/galo taku avaga ◯ 4. Mavae
4. Ko gata ifea au akoakoga	◯1. Seki akoga; ◯2. Akoga lasaga muamua; ◯ 3. Akoga lasaga lua;<br>◯ 4. Akoga maluga atu
5. Sitiseni Tuvalu	◯ 1. Ao; ◯ 2. Ikaai (Kafai ikaai, ko gata foki loa iei te faka-sautalaga)
6. Koga/fenua ne fanau iei koe	◯ 1.Funafuti;<br>◯ 2.Nanumea; ◯3.Nanumaga; ◯4.Niutao;<br>◯ 5.Vaitupu; ◯6. Nui; ◯7. Nukufetau;<br>◯ 8.Nukulaelae; ◯9. Niulakita<br>◯ 10.Others/ Immigrant
7. Ko fia nei tausaga ne nofo iei koe i te fenua/koga tenei e nofo koe iei?	__________ tausaga
8. Tulaga o tau galuega ite vaitau nei	◯ 1. Galue tumau;<br>◯ 2. See galue tumau; ◯ 3. Seki galue/koi akoga
9. Peofuga ite masina (Ko tusi te aofaki ite avanoaga tena ite sua feitu)	$_________ (AUD)

TAIWAN

**Vaega II: Masaki tutumau (Fakamolemole fili te “Ao” mafai se masaki ko leva; kafai seai, fili “Ikaai”; kafai see mautinoa tonu, fili “See mautinoa”)**	
10. Toto maluga	◯ 1.Ao; ◯ 2.Ikaai; ◯ 3. See mautinoa
11. Maluga te sinu i te foitino (Dyslipidemia)	◯ 1.Ao; ◯ 2.Ikaai; ◯ 3. See mautinoa
12. Sukaa	◯ 1. Ao ◯ 2. Ikaai; ◯ 3. See mautinoa

**Vaega III: Faifaiga masani ki mea tau meakai**				
13. E mata e kai fakafia ne koe meakai konei mai lalo ite tausaga ko teka atu? Pefea te uke o au mea ee kai i taimi taki tatasi? (**Fakamolemole ko tusi se napa i avanoaga kona mai lalo**)				
Meakai	Aofaki o meakai		Faka fia taimi	
	Saisi o tau tifa/ipu i taimi kai (1-8)	Fakafia taimi ne aasu/fakasoko iei tau tifa/ipu	Ite aso (0-3)	Aso i te masima (0-30)
Laisi				

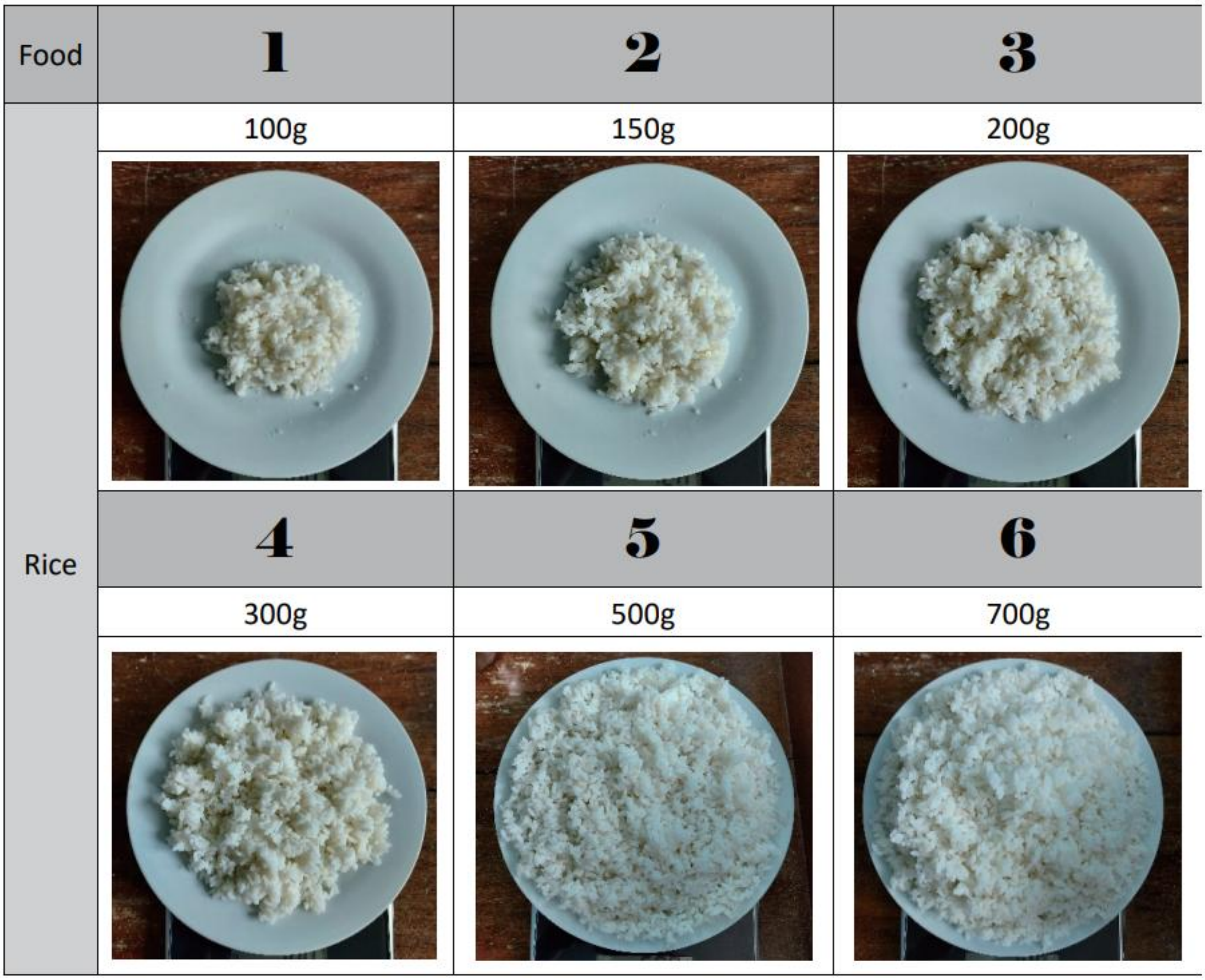

Nutolo (instant noodles)				

Food	1	2	3
Instant noodles	42.5g	85g	170g
	0.5 pc	1 pc	2 pcs
(raw)	×0.5	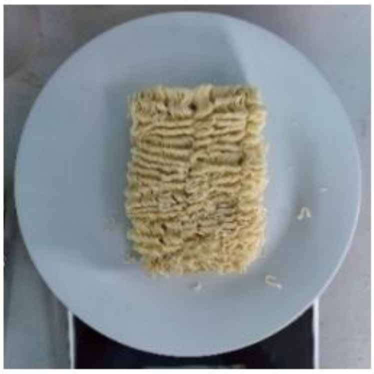	×2
(boiled)	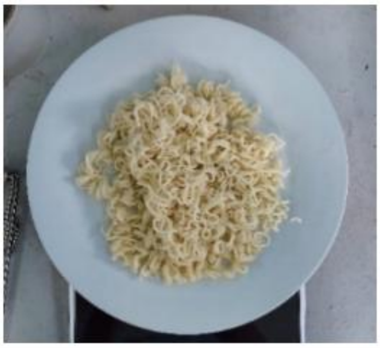	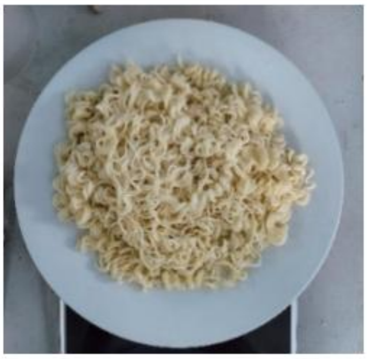	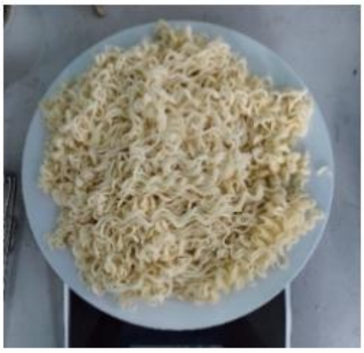

Masi				

Food	1
Breakfast crackers	10g
	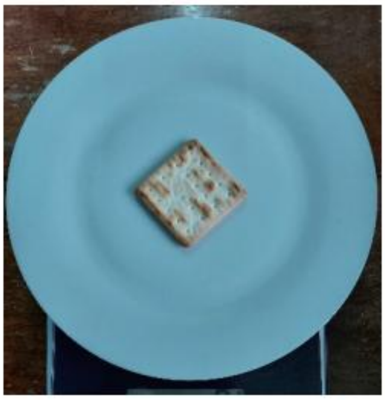

Talo/tapioca/mei

Food	1	2	3	4	5	6	7
swamp taro<br>taro<br>cassava<br>breadfruit	80g	160g	320g	400g	480g	560g	640g
	×0.5	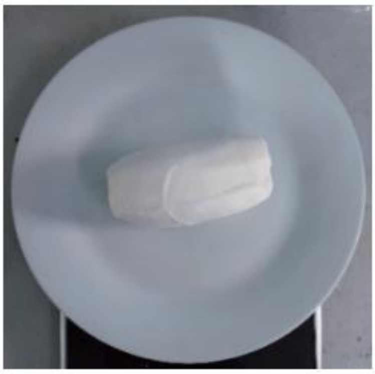<br>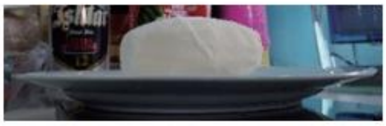	×2	×2.5	×3	×3.5	×4

Falaoa

Food	1
Toast	50g
	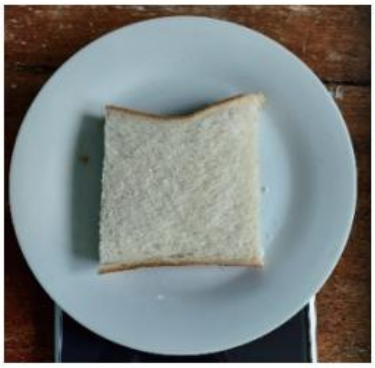

Ika

Food	1	2	3	4
Fish	100g	200g	300g	400g
	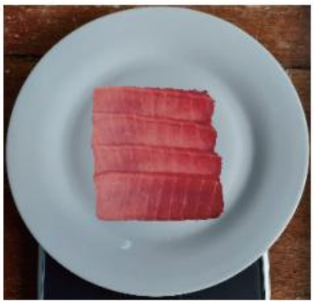	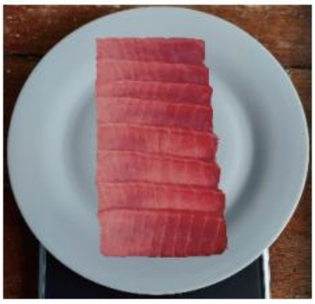	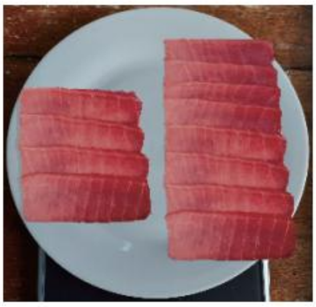	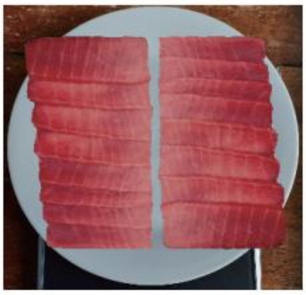

Moa				

Food	1	2	3	4
Chicken	70g	105g	140g	175g
	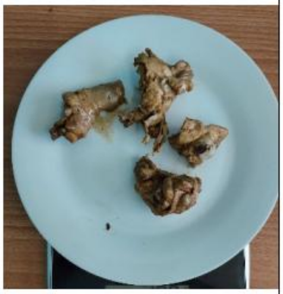	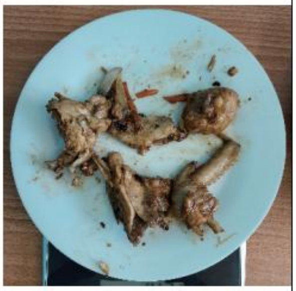	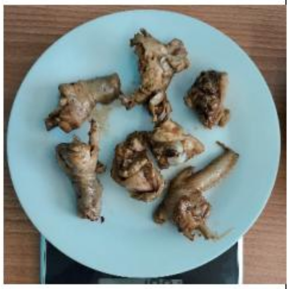	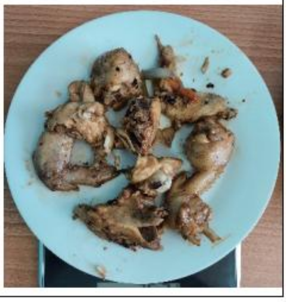
	5	6	7	
	210g	245g	280g	
	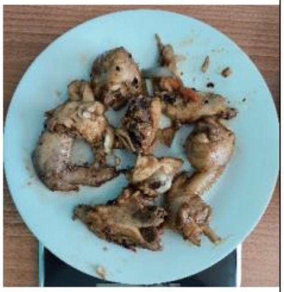	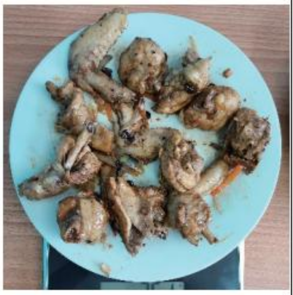	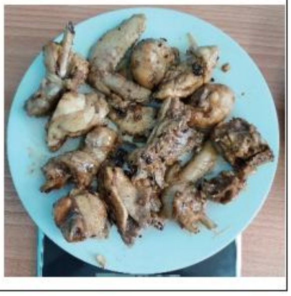	

Puaka/pulumakau/ mamoe				

Food	1	2	3
Pork/ Lamb/ Beef	70g	105g	140g
	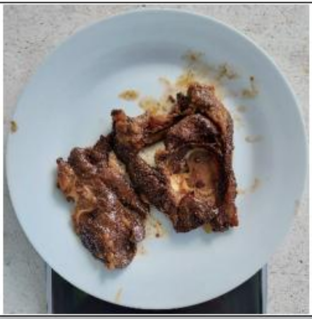	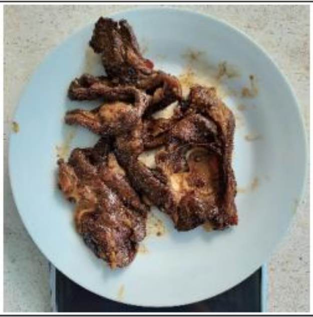	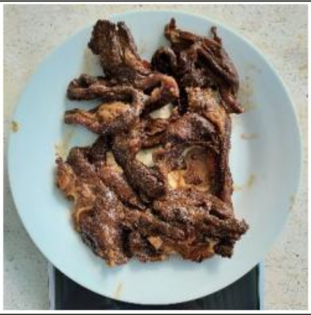
	4	5	
	175g	210g	
	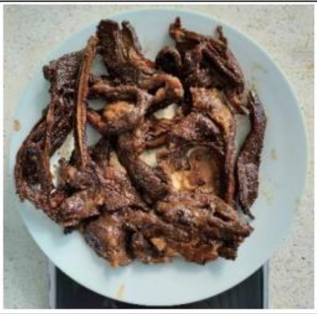	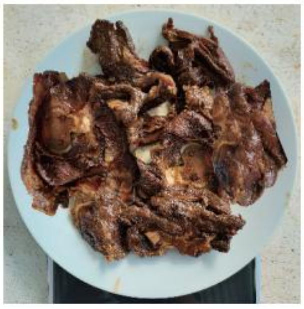	

Fuamoa				

Food	1	2	3
Egg	45g	90g	135g
	1 pc	2pcs	3pcs
(boiled)	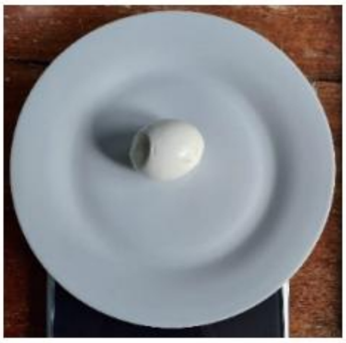	×2	×3
(stir-fried)	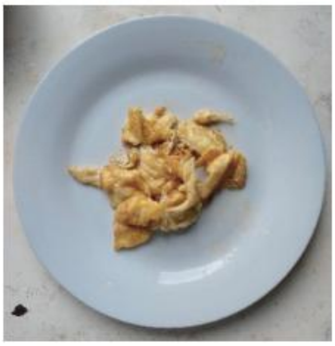	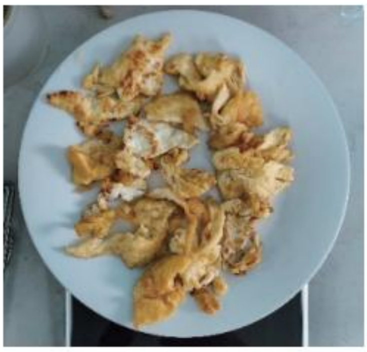	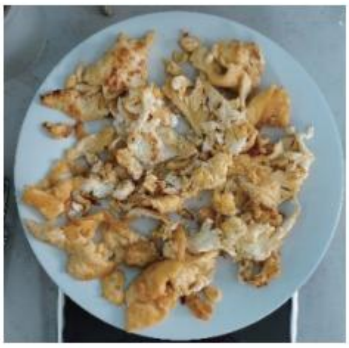

Kapisi				

Food	1	2	3	4
Cabbage	50g	100g	150g	200g
(raw)	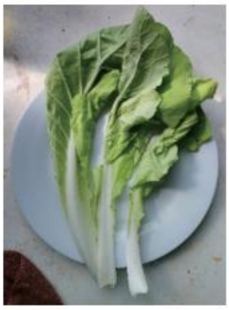	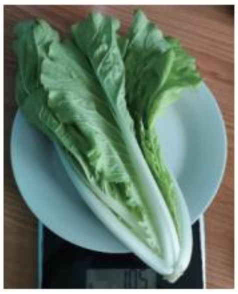	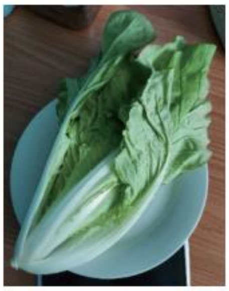	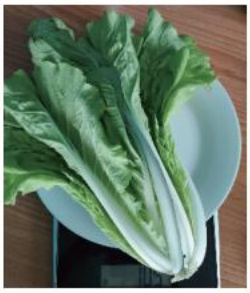
(stir-fried)	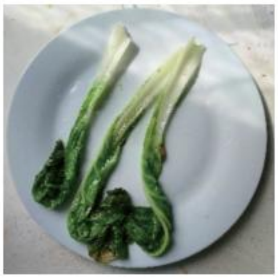	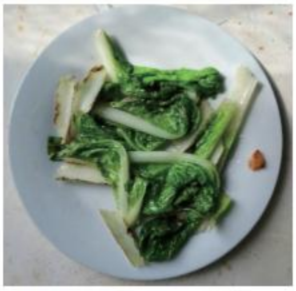	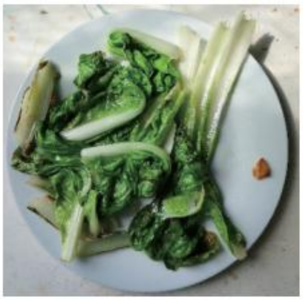	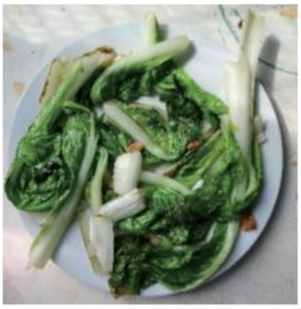

Kukama

Food	1	2	3
Cucumber	25g	50g	100g
	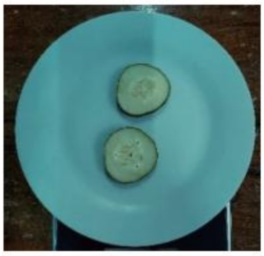	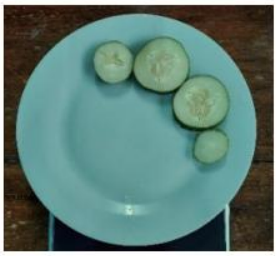	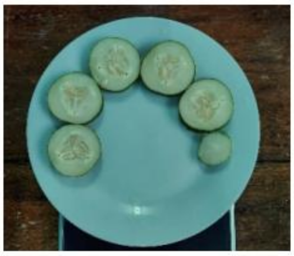
	4	5	6
	150g	200g	250g<br>half medium cucumber
	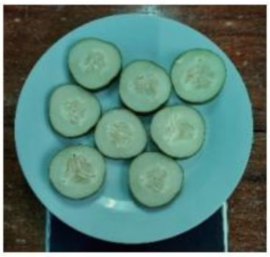	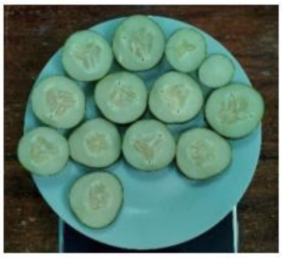	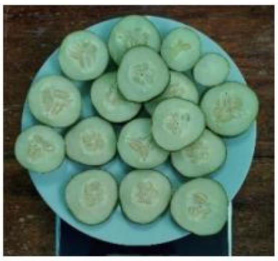

Vesiapolo togi i sitoa

Food	1	2	3
Imported vegetables	50g	100g	150g
	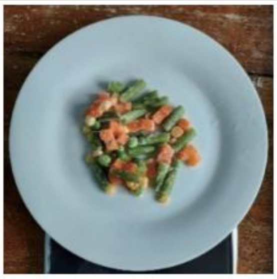	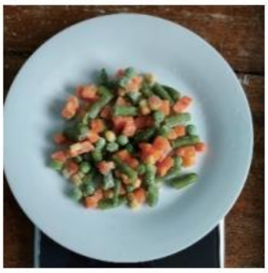	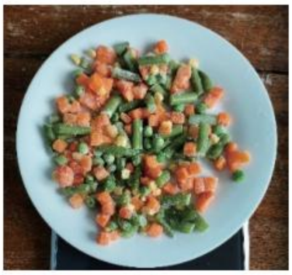
	4	5	
	200g	250g<br>half pack	
	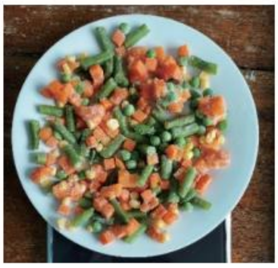	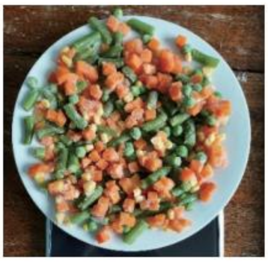	

Laulu

Food	1	2	3	4
Bird's-nest fern (Laurũ)	50g	100g	150g	200g
(raw)	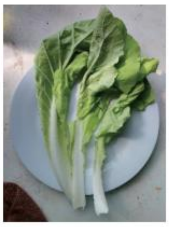	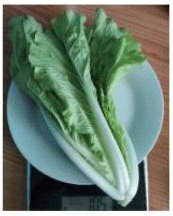	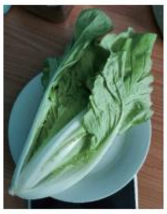	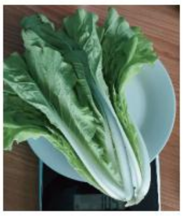
(stir-fried)	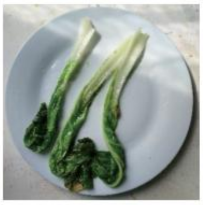	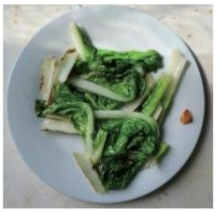	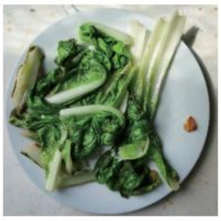	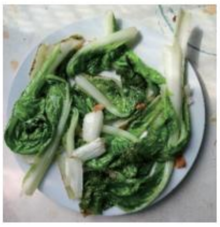

Futi

Food	1
Banana	70g
	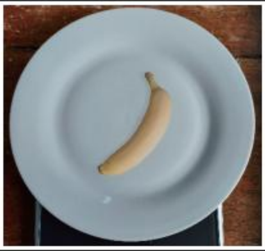

Olesi

Food	1	2	3	4
Papaya	100g	150g	200g	250g
	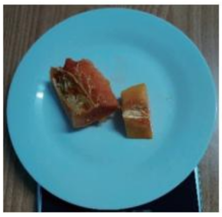	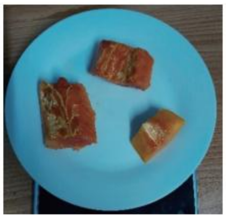	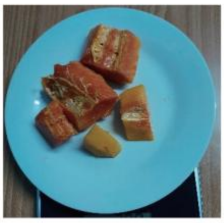	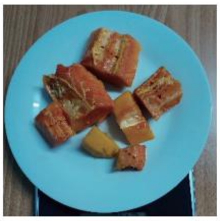
	5	6	7	
	300g	350g	400g	
	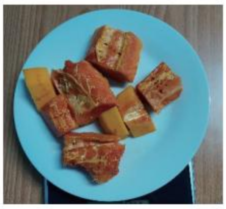	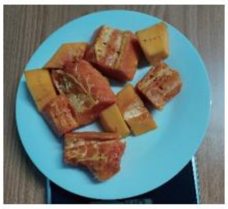	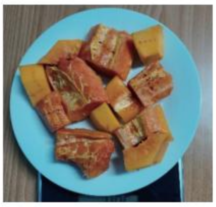	

TAIWAN CDF

Niu				

Food	1
Coconut	200g
	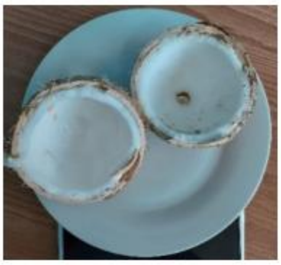

Fuaga lakau togi i sitoa (pela mo apolo, pea mo olenisi)				

Food	1
Imported fruits	130g
(apple)	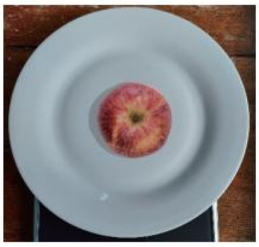
(orange)	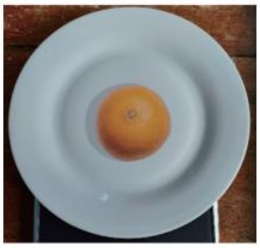

Meainu magalo (Pela mo meainu palu, Coke, Milo)				

Food	1	2	3	4
Sweetened drink (Juice, coke, milo, coffee)	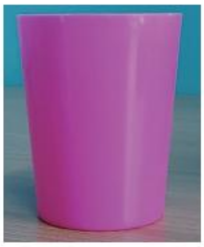	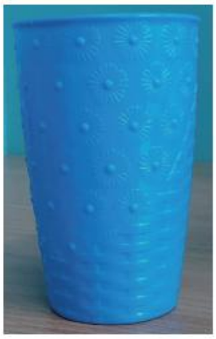	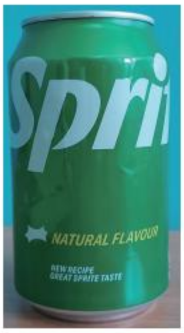	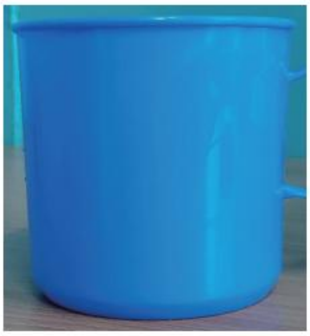

Susu				

Food	1	2	3
milk	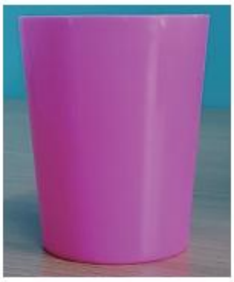	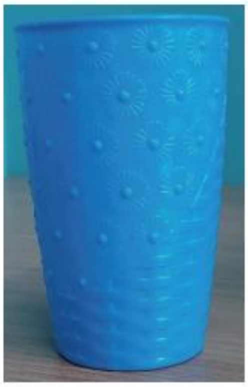	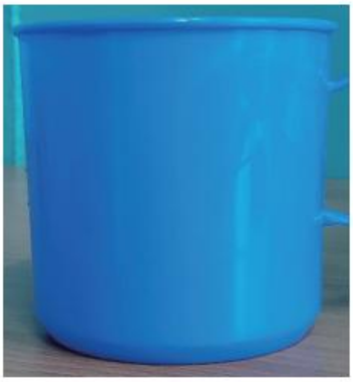

Meakai foliki (pela mo keke, masi keke, ice cream, chips, etc)				

Food	1	2	3
Delicates	10g	30g	50g<br>small size
	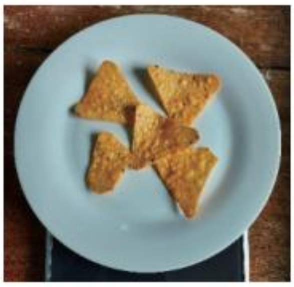	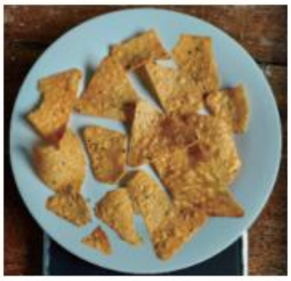	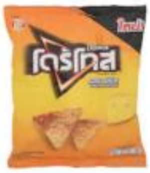
	4	5	6
	100g	200g	450g
	medium size	big size	party size
	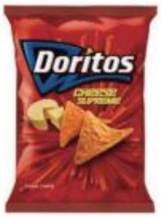	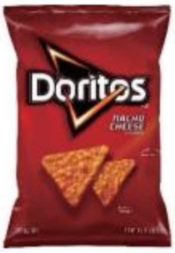	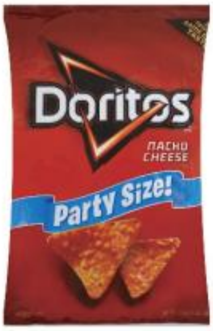

14. Kafai koe se tino e pusi, e mata e fakafia taimi e masani koe o pusi iei?	◯ 1.Aso katoa; ◯ 2. Nisi taimi/seasea loa ◯ 3. Au see pusi
15. E isi se tino i te otou kaiga e pusi?	◯ 1. Ao; ◯ 2. Ikaai
16. E fakafia taimi koe inu kamagi/meainu malosi i loto ite vaiaso (alcohol)?	◯ 1. Aso katoa; ◯ 2. Nai taimi fua; ◯ 3. Au seeti inu kamagi
17. E fakafia taimi e masani koe io me se tino i te otou kaiga o togi fuagalaku mo vesiapolo mai te fatoaga a te Kaupule?	◯ 1. Fakalua io me siliga atu ite vaiaso e tasi;<br>◯ 2. Fakafia taimi i te masina e tasi;<br>◯ 3. Fakatasi io me seai loa ite masina
18. E isi se fatoaga a te otou kaiga ite fale?	◯ 1. Ao; ◯ 2. Ikaai (Kafai seai, see taligina a fesili napa 19 ki te 22)
19. Ko fia nei te leva o te fakaaogaga ne te otou kaiga te fatoaga tena?	__________ tausaga
20. Pefea te lasi o te fatoaga?	________ fatoaga fakatuutuu io me ________sq. meters
21. Nea fuaga lakau/vesiapolo e toki ite fatoaga (nei io me se taimi ko teka atu)?<br>**(Mafai ne koe o fili e 2 io me siliga atu a tali)**	◯ 1. Bele; ◯ 2. 'Pepa (capsicum, chili);<br>◯ 3. Kapisi; ◯ 4. Kukama; ◯ 5. Tomato;<br>◯ 6. Panikeni;<br>◯ 7. Nisi mea aka (Pela mo niu, pulaka, futi, olesi)
22. Kooi a tino e paanaki saale mo latou te tokiga/tausiga o te fatoaga?	◯ 1. Koe totino; ◯ 2. Ou matua; ◯ 3. Au tama<br>◯ 4. Nisi tino o te kaiga

**Vaega IV: Fesili ki tulaga o te moe o koe (Fakamolemole tali ki ou mafaufauga totino)**	
23. Koe 'golo kii ma moe (pela, e lagona ne tino mai tua o te potu e moe koe iei)?	◯ 1. Ao; ◯ 2. Ikaai
24. Koe fiita saale io me vaivai tou foitino ite ao?	◯ 1. Ao; ◯ 2. Ikaai
25. E mata tou manavaga e fano lei loa i taimi e moe koe iei io me fakaitia nisi taimi?	◯ 1. Ao, e lei loa ; ◯ 2. Ikaai, e tai fakaitia saale nisi taimi

**Vaega V: Fesili ki luga i te fakamalosilosiga o foitino**	
26. I te vaiaso tenei ko teka, e fia aso ne fakamalosilosi iei tou foitino i polokalame tai faigata pela mo te sausau mea mafa (gym/weights), lakapi, tele, saka, volipoolo io me ko te 'kau? **<u>Se aofia iei te sasale</u>**.	__________aso ite vaiaso<br>◯ Seai ne polokalame fakamalosilosi foitino (See taligina te fesili)
27. E fia te leva o polokalame fakamalosilosi kona ne aofia koe iei?	_________ minute ite aso<br>◯ Seiloa tonu te leva

Fafetai mo tou taimi ne fakaavanoa mai!

TAIWAN ICDF

Community-Based Practice Survey on Nutrition in Tuvalu, year 2023

Interview Region	Interview Date	Height	Weight	Waist Circumference	Neck Circumference
◯1.Funafuti; ◯2.Nanumea;<br>◯3.Nanumaga; ◯4.Niutao;<br>◯5.Vaitupu; ◯6.Nui;<br>◯7.Nukufetau; ◯8.Nukulaelae;<br>◯9. Niulakita<br>◯10.Fetuvalu High School<br>◯11.Motufoua High School	(YYYYMMDD)	(cm)	(kg)	(cm)	(cm)

**Part I: Demographics**	
1. Sex	◯ 1. Male; ◯ 2. Female
2. Date of Birth (DD/MM/YYYY)	
3. Marital Status	◯ 1. Single; ◯ 2. Married;<br>◯ 3. Widow (Widower); ◯ 4. Divorced
4. Highest Education Level	◯1. None; ◯2. Elementary school;<br>◯ 3. High school; ◯ 4. College or above
5. Tuvaluan Citizenship	◯ 1. Yes; ◯ 2. No (Ends interview)
6. Which island were you born?	◯ 1.Funafuti;<br>◯ 2.Nanumea; ◯3.Nanumaga; ◯4.Niutao;<br>◯ 5.Vaitupu; ◯6. Nui; ◯7. Nukufetau;<br>◯ 8.Nukulaelae; ◯9. Niulakita<br>◯ 10.Others/ Immigrant
7. How long have you been living in the current island?	__________Year(s)
8. Work Status	◯ 1. Regular or fixed work;<br>◯ 2. Temporary work; ◯ 3. None or students
9. Monthly Income (Please fill in the blank)	__________ (AUD)

TAIWAN ICDF

**Part II: Past Medical History (Please select "Yes" if you have the following conditions; otherwise please select "No"; if not sure, please select "I don't know")**	
10. Hypertension (High blood pressure)	○ 1. Yes; ○ 2. No ; ○ 3. I don't know
11. Dyslipidemia (High blood fat)	○ 1. Yes; ○ 2. No ; ○ 3. I don't know
12. Diabetes (High blood sugar)	○ 1. Yes; ○ 2. No ; ○ 3. I don't know

Part III: Practice

13. How often do you consume the following foods during past year? What is the average amount of each consumption? (**Please write a number in each cell below**)

Items	Amount		Frequency	
	Portion size each time (1-8)	Number of portion(s) each time	Times per day (0-3)	Days per month (0-30)
Rice				
Instant noodles				
Breakfast crackers				
Swamp taro/ Taro/ Cassava/ Breadfruit				
Toast				
Fish				
Chicken				
Pork/ Lamb/ Beef				
Egg				
Cabbage				
Cucumber				
Imported vegetables				
Bird's-nest fern (Laurũ)				
Banana				
Papaya				
Coconut				
Imported fruits				
Sweetened drink (Ex: juice/ coke/ milo/ coffee)				
Milk				
Delicates (Ex: cake/ cookies/ ice cream/ chips)				

TAIWAN ICDF

14. How often do you smoke?	◯ 1. Everyday; ◯ 2. Occasionally/ socially; ◯ 3. No
15. Does your family member smoke?	◯ 1. Yes; ◯ 2. No
16. How often do you drink (alcohol)?	◯ 1. Everyday; ◯ 2. Occasionally/ socially; ◯ 3. No
17. How often do you or your family buy fruit and vegetable from government or kaupule garden?	◯ 1. More than once a week;<br>◯ 2. Several times each month;<br>◯ 3. Less than once each month
18. Does your family own a home garden?	◯ 1. Yes; ◯ 2. No (Skip questions 19-22)
19. How long has your family been using the garden?	__________Year
20. How large is the garden?	__________Gardening bed(s) or __________square meters
21. Which crops are/were raised in the garden (both now and before)? (**Multiple choices**)	◯ 1. Spinach; ◯ 2. Pepper (capsicum, chili);<br>◯ 3. Cabbage; ◯ 4. Cucumber; ◯ 5. Tomato;<br>◯ 6. Pumpkin;<br>◯ 7. Others (Ex. Coconut, swamp taro, banana, papaya)
22. Who are the main persons taking care of the garden in your family?	◯ 1. Yourself; ◯ 2. Parents; ◯ 3. Children<br>◯ 4. Other family members

**Part IV: Sleep questionnaire (Please respond according to your own thoughts)**	
23. Do you snore loudly (louder than talking or loud enough to be heard through closed doors)?	◯ 1. Yes; ◯ 2. No
24. Do you often feel tired, fatigued, or sleepy during daytime?	◯ 1. Yes; ◯ 2. No
25. Has anyone observed you stop breathing during your sleep?	◯ 1. Yes; ◯ 2. No

**Part V: International Physical Activity Questionnaire**	
26. During the **last 7 days**, on how many days did you do **moderate to vigorous** physical activities like heavy lifting, rugby, running, dancing, volleyball or swimming? **<u>Do not include walking</u>**.	__________days per week<br>◯ -9. No vigorous physical activities (Skip question 27)
27. How much time did you usually spend doing **moderate to vigorous** physical activities on one of those days?	__________minutes per day<br>◯ -9. Don't know/Not sure

Thank you for your time and cooperation!

TAIWAN

Appendix S2.

MINISTRY OF HEALTH

PRINCESS MARGARET HOSPITAL

PUBLIC HEALTH SERVICES DIVISION

PO Box 41, Fakaifou District, Funafuti, TUVALU. Reception Phone: (688) 20416 For Medical Emergency Dial: 999

Notification of Initial Study Exemption Determination

Date: June, 30th 2022

Principal Investigator and email: Yuan-Hung Lo; tm.tuvalu@icdf.org.tw

Protocol Title: Community-based nutrition knowledge, behavior and attitude survey in Tuvalu during COVID-19 pandemic

Principal Investigator: Yuan-Hung Lo

Funding Source: Taiwan International Cooperative and Development Fund

Ethical Review Date: 2/9/2022, Effective date: 2/9/2022

Review Action: Exempt

This initial study submission meets the criteria for exemption per the regulations found at the Declaration of Helsinki. As such, additional ethical review is not required. The Principal Investigator is responsible for ensuring compliance with any applicable local government or institutional laws, legislation, regulations and/or policies, whether conducting research internationally or nationally.

The determination that your research is exempt does not expire, and you will not file annual renewals. If changes to the research are proposed that would alter the original exemption determination, you should be submitted ethical review again.

If unsure, contact the Ministry of Health, Social Welfare and Gender Affairs. If you have any questions, please contact me at 688-20480.

Sincerely,

Vine. Sosene
Chief Public Health (Ag)
Tuvalu Ministry of Health

GOVERNMENT OF TUVALU · MINISTRY OF HEALTH · TUVALU·MO·TE·ATUA

cc: Acting Permanent Secretary of Health (Pelesala. Kaleia).
